# Knockdown of lncRNA AK139328 alleviates myocardial ischaemia/reperfusion injury in diabetic mice via modulating miR‐204‐3p and inhibiting autophagy

**DOI:** 10.1111/jcmm.13754

**Published:** 2018-07-25

**Authors:** Si‐yang Yu, Bo Dong, Zhen‐fei Fang, Xin‐qun Hu, Liang Tang, Sheng‐hua Zhou

**Affiliations:** ^1^ Department of Cardiovascular Medicine The Second Xiangya Hospital Central South University Changsha Hunan China

**Keywords:** diabetes mellitus, lncRNA AK139328, MiR‐204‐3p, myocardial ischaemia/reperfusion injury

## Abstract

This study was aimed at investigating the effects of lncRNA AK139328 on myocardial ischaemia/reperfusion injury (MIRI) in diabetic mice. Ischaemia/reperfusion (I/R) model was constructed in normal mice (NM) and diabetic mice (DM). Microarray analysis was utilized to identify lncRNA AK139328 overexpressed in DM after myocardial ischaemia/reperfusion (MI/R). RT‐qPCR assay was utilized to investigate the expressions of lncRNA AK139328 and miR‐204‐3p in cardiomyocyte and tissues. Left ventricular end diastolic diameter (LVEDD), left ventricular end systolic diameter (LVESD), left ventricular ejection fraction (LVEF) and fractioning shortening (FS) were obtained by transthoracic echocardiography. Haematoxylin‐eosin (HE) staining and Masson staining were utilized to detect the damage of myocardial tissues degradation of myocardial fibres and integrity of myocardial collagen fibres. Evans Blue/TTC staining was used to determine the myocardial infarct size. TUNEL staining was utilized to investigate cardiomyocyte apoptosis. The targeted relationship between lncRNA AK139328 and miR‐204‐3p was confirmed by dual‐luciferase reporter gene assay. MTT assay was used for analysis of cardiomyocyte proliferation. Western blot was utilized to investigate the expression of alpha smooth muscle actin (α‐SMA), Atg7, Atg5, LC3‐II/LC3‐I and p62 marking autophagy. Knockdown of lncRNA AK139328 relieved myocardial ischaemia/reperfusion injury in DM and inhibited cardiomyocyte autophagy as well as apoptosis of DM. LncRNA AK139328 modulated miR‐204‐3p directly. MiR‐204‐3p and knockdown of lncRNA AK139328 relieved hypoxia/reoxygenation injury via inhibiting cardiomyocyte autophagy. Silencing lncRNA AK139328 significantly increased miR‐204‐3p expression and inhibited cardiomyocyte autophagy, thereby attenuating MIRI in DM.

## 1 INTRODUCTION

The blockage of blood vessels in the heart, namely myocardial ischaemia, is among the most prevalent causes of worldwide mortality.^1^ The reperfusion therapy with blood flow restoration is the recommended treatment for infarct recovery, yet is more curative for ischaemia of short duration.^1^, ^2^ Moreover, reperfusion paradoxically leads to additional damage to ischaemic myocardium.^2^, ^3^ Diabetic patients are at higher risk of ischaemic heart disease as well as myocardial ischaemia/reperfusion (I/R) injury (MIRI).^4^ Moreover, researchers have revealed that diabetes mellitus aggravates MIRI and influences the functioning of ischaemic preconditioning and some cardioprotective pharmacologic agents.^5^ Therefore, the investigation of pathological mechanism of diabetes in interaction with MIRI novel targets would be contributing to diabetic myocardium protection.

Non‐coding RNAs including microRNAs (about 20–22 nt) and long non‐coding RNAs (lncRNAs, >200 nt) have already been heated research topics.^6^ Previous researches have suggested that some lncRNAs were closely associated with myocardial ischaemia‐reperfusion. Li et al provide evidence for lncRNA KCNQ1OT1 knockdown being a preventive solution to MIRI.^7^ Zhao et al demonstrated that lncRNA MALAT1 was involved in myocardial ischaemia‐reperfusion and down‐regulation of MALAT1 contributed to the reduction of cardiomyocyte apoptosis.^8^ LncRNA AK139328, which has been indicated by Chen et al to be involved in liver I/R injury, is a vital member of lncRNAs.^9^ However, the mechanism and role of lncRNA AK139328 in MIRI have not been completely investigated.

Apoptosis or type I programmed cell death, a main type of cell death that occurs when DNA damage is irreversible, is characterized by distinct morphological and biochemical changes.^10^, ^11^ I/R induced myocardium apoptosis, which becomes a key factor in MIRI pathogenesis.^12^ Type II programmed cell death, namely autophagy, is by mechanism distinct from apoptosis.^13^ Reduced superoxide dismutase (SOD) and increased lactate dehydrogenase (LDH) and malondialdehyde (MDA) concentration appeared in line with autophagy.^14^ Recent studies emphasized the dynamic process of autophagy, that is autophagic flux in understanding the impairment caused by MIRI.^15^ In addition, while basal autophagy maintains cell recycling and thereby inhibits cell apoptosis in infarct area, excessive acceleration of autophagy would probably lead to an aggravation in MIRI.^11^, ^15^ A deep investigation into the apoptotic and autophagic mechanism underlying MIRI is of pivotal importance in the advancement of myocardial ischaemia treatment.

Recent studies have also suggested that various miRNAs could regulate cell apoptosis, such as miR‐1, miR‐133, miR‐208, miR‐21 and miR‐204. MiR‐204 has already been identified to exert anti‐autophagy effects and may also regulate metabolic recovery in MIRI.^16^ Nonetheless, the mechanisms of how such miRNAs regulate myocardial cell apoptosis and autophagy in MIRI as modulated by lncRNAs remain unknown. Understanding the influence of their interaction on autophagy in MIRI will contribute to develop new diagnostic and therapeutic strategies.

In this study, we constructed myocardial I/R models in normal mice (NM) and diabetic mice (DM). The expression level of AK139328 and miR‐204‐3p in cardiomyocyte and tissues was detected, along with their targeting relationship. After AK139328 silencing, the apoptosis ratio and the autophagy level of cardiomyocyte were evaluated. Tissue damage and infarct size were determined by staining. Our study may supply novel therapeutic strategies for the treatment of MIRI.

## 2 MATERIALS AND METHODS

### 2.1 Experimental animals and treatments

Adult healthy male C57BL/KsJ db/+ mice (6‐8 weeks), weight 20‐25 g, and adult male C57BL/KsJ db/db mice were purchased from Cavens Lab Animal Co., Ltd. (Changzhou, China) and kept in a temperature‐controlled room with a constant temperature of 26 ± 2°C, a humidity of 60%‐80% and a programmed 12‐hours light/12‐hours dark cycle for circadian control. Fasting for one day was requested before the experiments. The mice were kept at specific pathogen‐free (SPF) level to prevent the infection of specific disease. C57BL/KsJ db/+ mice were used as normal mice, and C57BL/KsJ db/db mice were used as diabetic mice. The biological characteristics of C57BL/KsJ db/+ and C57BL/KsJ db/db mice are listed in Table S1. C57BL/KsJ db/+ were randomly divided into 2 groups: the sham (normal mice, NM) group and the I/R (NM) group. C57BL/KsJ db/db mice were randomly assigned to 4 groups: the sham (DM) group, the I/R (diabetic mice, DM) group, the I/R+Lv‐shRNA1‐AK139328 group and the I/R+Lv‐shRNA2‐AK139328 group. Each of these 6 groups contained 40 mice. Animal protection measures and all experimental procedures were strictly implemented following the agreement of the animal experiment regulations approved by the Second Xiangya Hospital, Central South University.

### 2.2 Myocardial ischaemic reperfusion injury (MIRI) surgery

According to the explanation on myocardial I/R model construction by Gao et al,^17^ all mice were anesthetized with inhaled ether (3 minutes) and fixed for endotracheal intubation by the use of a small animal respirator. A longitudinal incision was made from the third to fourth ribs, exposing the heart. Then, a 5‐0 Prolene suture was placed around the 2 cm of the root of left anterior descending coronary artery (LAD). The suture was loosened after occlusion for 30 minutes, which was followed by 120‐minutes reperfusion of LAD.

### 2.3 Microarray analysis

Mouse LncRNA Expression Array V3.0 chip (Aksomics, Shanghai, China) was utilized to screen differentially expressed lncRNAs in NM and DM after MIRI. Total lncRNAs were extracted for chip hybridization. The expression levels of lncRNAs were represented by fluorescence intensity values. The differential expression criteria were set as fold change >2, *P* < .05.

### 2.4 RT‐qPCR assay

TRIzol™ reagent (Invitrogen, Carlsbad, CA, USA) was used to obtain total RNA. Two microgram of total RNA was used for reverse transcription by PrimeScript® 1st Strand Synthesis Kit (TaKaRa,Tokyo, Japan). The real‐time RT‐qPCR was performed with QuantiTect SYBR® Green RT‐PCR Kit (QIAGEN, Dusseldorf, Germany). The primer sequences for RT‐qPCR are shown in Table S2. Normalization was performed with GAPDH (for lncRNAs) or U6 (for miRNA). The relative expression of lncRNA AK139328/AK028326/Aasdh/Slco6d1/Malat1/Eif4a2/Gomafu/Vps13d and miR‐204‐3p was determined with 2^−▵▵*ct*^ method.

### 2.5 Construction of lentiviral vectors for shRNA‐AK139328

Specific short hairpin RNA (shRNA1 and shRNA2) of mouse AK139328 and scramble oligonucleotides were synthesized by GenePharma (Shanghai, China) (Table S3). A unit of 20 μg of AK139328 shRNA1, AK139328 shRNA2 or scrambled shRNA was constructed into BLOCK‐iT™ lentiviral RNAi expression system (Invitrogen). Briefly, 293 FT cells (Thermo Fisher, Shanghai, China) were cotransfected with the lentiviral vectors and packaging vectors. The supernatant was collected after 48 and 72 hours. The viral supernatant was concentrated with the lentivirus concentration reagent (Biomiga, CA, USA). High titre virus (1 × 10^9^ PFU/mL) was resuspended in PBS. Narcosis was performed with 2% isoflurane, and to expose the heart, an incision between the fourth and fifth left ribs was made. A volume of 10 μL concentrated lentivirus with AK139328 shRNA was delivered via intramyocardial injection into the apex and anterolateral wall with a 30‐gauge needle. Another group of mice injected with 10 μL of lentivirus‐scrambled shRNA was regarded as NC group.

### 2.6 Myocardial enzyme determination

Mice were anesthetized with inhaled ether 24 hours after reperfusion. Blood sampled from the inner canthus vein was centrifuged for 15 minutes. The serum was kept at a temperature of −20°C. Semiautomatic biochemical analyser was used to assess the expression of phosphocreatine kinase (CK), creatine kinase MB (CK‐MB) and lactate dehydrogenase (LDH) in the serum.

### 2.7 Echocardiographic assessment

At the end of reperfusion, mice were re‐anesthetized with isoflurane, fixed on the experiment table and studied on an echocardiography system (Sequoia Acuson, 15‐MHz linear transducer; Siemens, Erlangen, Germany). The M curve was measured at the long axis of papillary muscle and left ventricle section level. The following variables were measured and averaged during 3 consecutive cardiac cycles: left ventricular end systolic diameter (LVESD), left ventricular end diastolic diameter (LVEDD), left ventricular end systolic volume (LVSV) and left ventricular end diastolic volume (LVDV). The left ventricular ejection fraction (LVEF) and left ventricular fractional shortening (LVFS) values were converted by the Simpson method with the following formula: LVEF = (LVDV − LVSV)/LVDV × 100%; LVFS = (LVDD − LVSD)/LVDD × 100%. LVEF and LVFS were used as parameters indicating cardiac function. The experiment was conducted 3 times, and the mean value was obtained.

### 2.8 Histopathology examination

The histopathology examination of myocardial tissues was performed with haematoxylin‐eosin (HE) staining. The left ventricle of heart samples was put in 10% formaldehyde solution, dehydrated in ethanol gradient, embedded in paraffin and cut down into slices of 4 μm. After deparaffinage, the samples were stained with haematoxylin and eosin. Then, the slices were mounted and observed under a light microscope (Leica Microsystems, Wetzlar, Germany).

### 2.9 Masson staining

Mice cardiac tissues were fixed with 10% formaldehyde for 24 hours at room temperature, then decalcified, dehydrated, permeabilized with xylene, embedded in wax and finally sliced into 5‐μm‐thick sections with a microtome. Wiegert's iron haematoxylin solution (Sigma‐Aldrich, St. Louis, MO, USA) was used to dye the cell nucleus for 5 minutes. Following rinsing with distilled water 3 times, the sections were stained with 0.7% Masson‐Ponceau‐acid fuchsin staining solution (Sigma‐Aldrich) for 10 minutes. Samples were then rinsed in 2% glacial acetic acid and differentiated in phosphomolybdic acid for 4 minutes. The sections were directly stained with 2% aniline blue dye solution (Sigma‐Aldrich). Following dehydrating with ethanol series, clearing with xylene and mounting with neutral resins, images of the stained sections were captured with a light microscope.

### 2.10 Infarct size determination

To prove the effects of I/R, the infarct size of the hearts was tested in this study. After the haemodynamic testing, the LAD was sutured. Evans Blue dye (1 mL of a 2.0% solution) was injected through a carotid artery catheter into the coronary circulation to delineate the *in vivo* area at risk. The heart was rapidly excised and frozen at a temperature of −20°C for 30 minutes and then serially cross‐sectioned in 1‐mm‐thick sections which were then incubated in pH7.4 1.0% 2,3,5‐triphenyl tetrazolium chloride (TTC) formulated in phosphate‐buffered solution (PBS) for 15 minutes at 37°C. Sizes of the normal region (area not at risk, ANAR), the ischaemic region (area at risk, AAR) and the infarct area (INF) were assessed by Image‐Pro Plus software, results of which were taken as INF/AAR × 100%.

### 2.11 Western blot assay

RIPA lysate (Beyotime, Shanghai, China) was used to obtain total proteins, 100 μg of which were segregated with SDS‐polyacrylamide gel electrophoresis and transferred onto polyvinylidene difluoride (PVDF) membranes. TBST containing 5% skim milk was used for membrane incubation for 1 hour. Then, the membranes experienced incubation with primary antibodies including anti‐SMA (Sigma‐Aldrich), anti‐Apg7 (ab52472, 1/100 000, Abcam, Cambridge, MA, USA), anti‐APG5L/ATG5 (ab108327, 1/1000, Abcam), anti‐LC3A/B (ab128025, Abcam), anti‐SQSTM1/p62 (ab56416, Abcam) and anti‐GAPDH (ab181603, 1:10 000, Abcam) at 4°C overnight. The membranes were washed in TBST 3 times and incubated with anti‐rabbit IgG H&L (HRP) secondary antibody (ab6721, 1:2000, Abcam) at room temperature for 1.5 hours. After washed with TBST thrice, the membranes were subjected to colour reaction by ECL Plus from Life Technology, and GAPDH was detected as control groups.

### 2.12 TUNEL staining

The heart was rapidly excised after reperfusion and sectioned into 5‐μm‐thick sections. Cardiomyocyte apoptosis was assessed in the heart sections by terminal deoxynucleotidyl transferase‐mediated dUTP nick‐end‐labelling (TUNEL) staining. TUNEL mix contained 50 μL enzyme solution and 450 μL label solution. Heart sections were incubated with 50 μL TUNEL mix at 37°C for 1 hour. The sections were washed in phosphate‐buffered solution (PBS) thrice and stained with DAPI and α‐actin. After washing with PBS for another 3 times, the sections were observed by fluorescence microscopy. Apoptosis ratio was taken as apoptosis cell number (green)/total cell number (blue) × 100%.

### 2.13 Caspase‐3 activity assay

Caspase‐3 activities were assessed by caspase‐3 activity assay kit (BestBio, Shanghai, China). The cardiomyocyte were lysed with 90 μL lysis buffer. Subsequently, 10 μL Ac‐DEVD‐ρNA was added to cell lysates, and the mixtures were incubated for 2 hours at room temperature. The absorbance at 405 nm of apoptosis cells and control cells was determined by enzyme immunoassay detector. Caspase‐3 activity was taken as the ratio of these 2 absorbance values.

### 2.14 Dual‐luciferase reporter gene assay

Wild‐type (WT) and mutated (MUT) 3′‐UTR of AK139328 was generated by PCR. The sequences after amplification were linked with Pmir‐GLO dual‐luciferase miRNA target expression vectors (Promega, Madison, WI, USA). HEK293T cells (BeNa Culture Collection, Shanghai, China) were cultured at 37°C with 90% high‐glucose DMEM (4 μL glutamine‐sodium pyruvate), 10% FBS and 5% CO_2_. After seeded onto 24‐well plates, the cells were cotransfected with miR‐204‐3p mimics or control mimics.

### 2.15 Cell isolation and culture

Cardiomyocytes were isolated from adult C57BL/KsJ db/+ mice heart. In brief, mice were narcotized with 2% isoflurane, and hearts were taken off and perfused at 37°C for 3 minutes with a Ca^2+^‐free bicarbonate‐based buffer. Enzymatic digestion was initiated by adding collagenase type B/D to the perfusion solution (R&D Systems, Minneapolis, MN, USA). After suitable digestion, 50 mmol/L Ca^2+^ was appended to the enzyme solution. Approximately 8 minutes later, the left ventricle was removed and further digested for 15 minutes at 37°C. The supernatant containing the dispersed myocytes was percolated into a sterile culture tube and centrifuged at 900 *g* for 2 minutes. The cell pellet was then resuspended in bicarbonate‐based buffer containing 125 mmol/L Ca^2+^. After the myocytes were pelleted by gravity for 10 minutes, the supernatant was aspirated, and the myocytes were resuspended in bicarbonate‐based buffer containing 250 mmol/L Ca^2+^. One hour after plating, cells were washed with PBS and non‐adhering cells were removed from the culturing system. Cells were randomized to receive into 2 groups: (i) control group (culture medium containing 5 mmol/L glucose) and (ii) high‐glucose group (culture medium containing 33 mmol/L glucose).

### 2.16 Hypoxia/reoxygenation (H/R) injury model

By the model of hypoxia/reoxygenation (H/R) injury, we analysed hypoxia‐induced and reoxygenation‐induced cell death. The cardiac myocytes were cultured under a hypoxic gas mixture supplemented with 95% N_2_ and 5% CO_2_, and the medium was placed in a hypoxic incubator (95% N_2_ and 5% CO_2_) for 3 hours which was followed by reoxygenation incubator (95% O_2_ and 5% CO_2_) for 3 hours. Cells of control group were cultured with 5% CO_2_ at 37°C for 6 hours.

### 2.17 Construction of shRNA plasmid vector

Interference sequences of lncRNA‐AK139328 were designed by BLOCK‐iT^TM^ RNAi designer (Invitrogen). Relevant sequences are shown in Table S3. BamH Ι and Hind III restriction sites were led into 3′‐UTR and 5′‐UTR of shRNA to synthetize oligonucleotide single strand. shRNA was linked with pGFP‐V‐RS plasmid vector. The plasmids were extracted after amplification.

### 2.18 Cell transfection

MiR‐204‐3p mimics and anti‐miR‐204‐3p inhibitor were obtained from GenePharma Technology Co., Ltd (Shanghai, China). Cells were transfected with the constructed shRNA1‐AK139328 plasmid vectors, miR‐204‐3p mimics and anti‐miR‐204‐3p inhibitor. Lipofectamine™ 3000 (Life Technologies, USA) was used for transfection. After transfection, all cells were collected for further experiments.

### 2.19 LDH, SOD and MDA determination

After cardiomyocyte H/R model establishment, the expression level of LDH was detected with LDH cytotoxicity assay kit (Beyotime, Shanghai, China). The expression level of SOD was detected with total superoxide dismutase assay kit (Solarbio, Beijing, China), and the expression level of MDA was detected with malondialdehyde assay kit (Solarbio). The level of MDA was taken as the disparity of the absorbance at 532 nm and 600 nm.

### 2.20 MTT assay

Myocardial cells were seeded onto 96‐well plates at a density of 1000‐10 000 cells/well and cultured in 200 μL cell culture medium at 37°C for 3‐5 days. Ten microlitre MTT (5 mg/mL, pH = 7.4, prepared with PBS) was added to culture the cells for 4 hours. After the medium was turned away, the precipitate was made soluble in 100 μL DMSO. An enzyme‐linked immunosorbent plate reader was utilized to determine the absorbance of each well.

### 2.21 Statistical analysis

The cell experiments were performed at least 3 times. The animal experiments were performed at least 6 times. The data were exhibited as mean ± SD. Student's *t* test was utilized to evaluate data between 2 groups, while the comparison among multiple groups was conducted by one‐way ANOVA. Statistical analysis was performed with GraphPad Prism 6.0 software. Significance level was set as *P* < .05.

## 3 RESULTS

### 3.1 Knockdown of lncRNA AK139328 suppressed the expression of CK, CK‐MB and LDH

**Figure 1:**
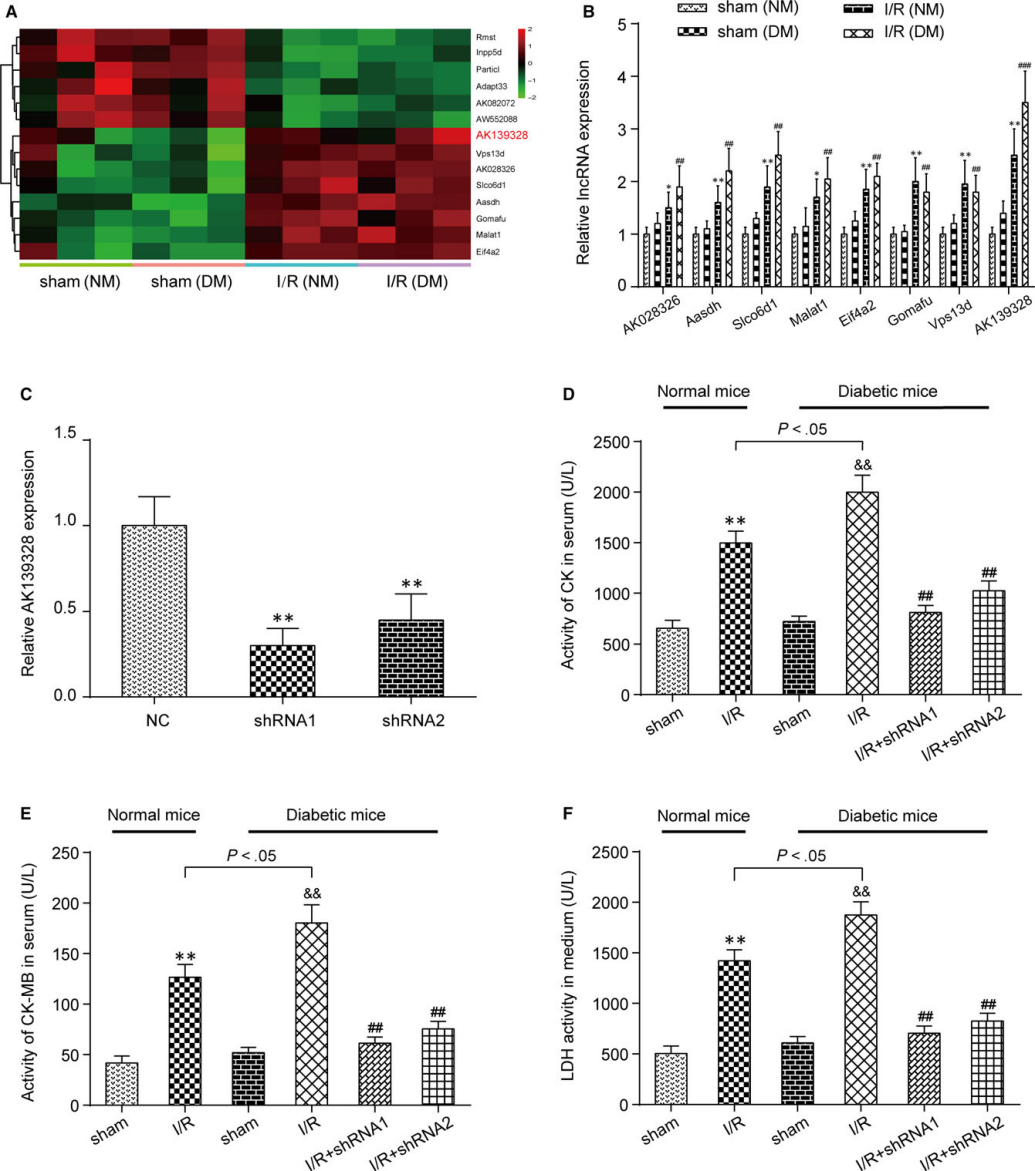
Knockdown of lncRNA AK139328 suppressed CK, CK‐MB and LDH expressions. A, The results of chip analysis showed the differentially expressed lncRNAs in normal mice (NM) and diabetic mice (DM) after myocardial ischaemia/reperfusion (MI/R). B, The expression of lncRNA AK139328 was indicated to be most up‐regulated by RT‐qPCR. *P < .05, **P < .01, compared with sham (NM) group. ^##^ *P* < .01, ^###^ *P* < .001, compared with sham (DM) group. C, The expression of lncRNA AK139328 in shRNA‐AK139328 group was inhibited. **P < .01, compared with NC group. D, The expression of CK after inhibiting AK139328 in DM was reduced significantly. **P < .01, compared with sham (NM) group, ^&&^ *P* < .01, compared with sham (DM) group, and ^##^ *P* < .01, compared with I/R (DM) group. E, The expression of CK‐MB after inhibiting AK139328 in DM was reduced significantly. **P < .01, compared with sham (NM) group, ^&&^ *P* < .01, compared with sham (DM) group, and ^##^ *P* < .01, compared with I/R (DM) group. F, The expression of LDH after inhibiting AK139328 in NM and DM was reduced significantly. **P < .01, compared with sham (NM) group, ^&&^ *P* < .01, compared with sham (DM) group, and ^##^ *P* < .01, compared with I/R (DM) group

As shown in Table S1, the blood glucose and serum insulin levels of C57BL/KsJ db/db mice were significantly greater than those of C57BL/KsJ db/+ mice. So, C57BL/KsJ db/+ mice were used as normal mice, and C57BL/KsJ db/db mice were used as diabetic mice. Then, differentially expressed lncRNAs in normal mice (NM) and diabetic mice (DM) after MIRI were selected with LncRNA Microarray v3.0 chip analysis (Figure 1A). The results of RT‐qPCR assay showed that lncRNA AK139328 was most up‐regulated in DM compared with other lncRNAs (Figure 1B). shRNA1 and shRNA2 were transfected into heart tissues of DM to inhibit the expression of lncRNA AK139328. The results of RT‐qPCR indicated that AK139328 was significantly down‐regulated compared with NC group, indicating successful transfection (Figure 1C). The expression of CK after I/R was higher in DM than that in NM, whereas knockdown of lncRNA AK139328 significantly suppressed the expression of CK (Figure 1D). The expression of CK‐MB after I/R was higher in DM than that in NM. Knockdown of lncRNA AK139328 repressed the expression of CK‐MB significantly in comparison with the I/R group (Figure 1E). Likewise, the expression of LDH after I/R was higher in DM in comparison with NM, while knockdown of lncRNA AK139328 marked a significant decrease in concentration of LDH (Figure 1F). The above results indicated that ischaemia in myocardium was alleviated by down‐regulated AK139328.

### 3.2 Knockdown of lncRNA AK139328 decreased cardiac function in DM

**Figure 2:**
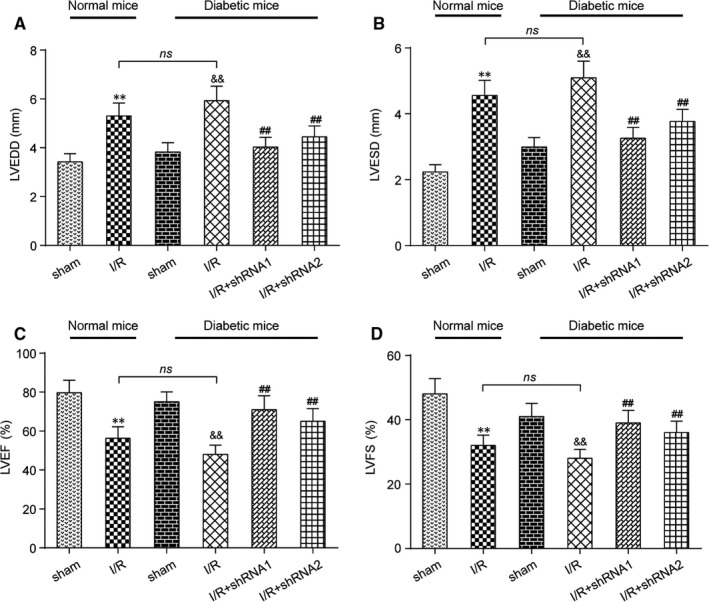
Knockdown of lncRNA AK139328 decreased cardiac function in diabetic mice (DM). A, Left ventricular end diastolic diameter (LVEDD) was significantly enlarged after MI/R in normal rats and diabetic rats, and silencing of AK139328 decreased LVEDD in diabetic rats. B, Left ventricular end systolic diameter's (LVESD) change in different groups was the same as LVEDD's. C and D, Left ventricular ejection fraction (EF) and left ventricular fractional shortening (FS) were reduced after MI/R in normal rats and diabetic rats, while down‐regulation of AK139328 restored EF and FS to control levels. **P < .01, compared with sham (NM) group, ^&&^ *P* < .01, compared with sham (DM) group, and ^##^ *P* < .01, compared with I/R (DM) group

Cardiac function was assessed by echocardiography in mice after MI/R injury. Left ventricular end diastolic diameter (LVEDD) and left ventricular end systolic diameter (LVESD) were significantly enlarged after I/R practice in NM and DM. Knockdown of lncRNA AK139328 repressed LVEDD and LVESD significantly in comparison with the I/R group (Figure 2A,B). Left ventricular ejection fraction (LVEF) and left ventricular fractional shortening (FS) were reduced after I/R treatment in NM and DM, while silencing of AK139328 restored LVEF and FS to normal levels, suggesting a protective role of shRNA‐AK139328 in DM suffering from MI/R injuries (Figure 2C,D).

### 3.3 Knockdown of lncRNA AK139328 relieved myocardial ischaemia/reperfusion injury in DM

**Figure 3:**
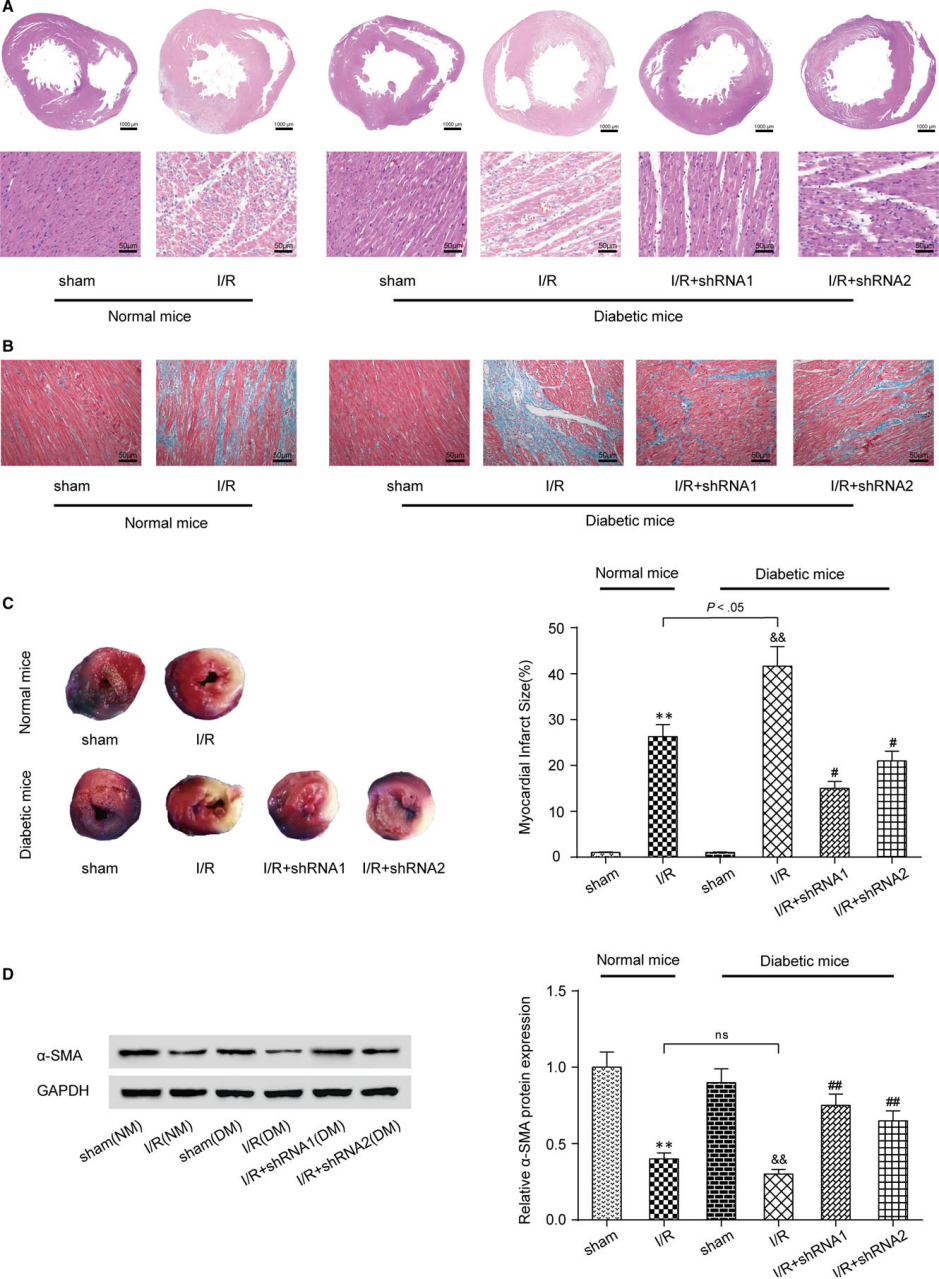
Knockdown of lncRNA AK139328 relieved myocardial ischaemia/reperfusion injury in diabetic mice (DM). A, The results of HE staining showed that knockdown of lncRNA AK139328 relieved the dreadful effects of I/R on myocardial tissue. B, Masson staining results displayed I/R that caused myocardial fibres generated a disorder arrangement and large amounts of collagen deposition. The myocardial collagen fibres were stained blue, and the myocardial fibres were stained red. C, Evans Blue/TTC staining results showed that I/R could lead myocardial infarction to normal mice (NM) and DM. The myocardial infarct size was reduced in DM on account of low expression of lncRNA AK139328. D, The expression of α‐SMA in MI/R groups was inhibited compared with sham groups. The knockdown of lncRNA AK139328 significantly improved the level of α‐SMA. **P < .01, compared with sham (NM) group, ^&&^ *P* < .01, compared with sham (DM) group, and ^#^ *P* < .05, ^##^ *P* < .01, compared with I/R (DM) group

Haematoxylin‐eosin (HE) staining results revealed the degree of myocardial tissue impairment in mice models. In Figure 3A, clear damage to myocardium could be observed in both normal and diabetic mice underwent I/R surgery in relation to sham groups, as was illustrated by increased blue‐purple stained areas. Besides, more obvious damage was found in DM compared to NM. For further‐grouped diabetic mice, knockdown of lncRNA AK139328 relieved the effects of I/R on myocardial tissue (Figure 3A). The myocardial sections were colourized with Masson staining and observed under a light microscope. The myocardial collagen fibres were stained blue, and the myocardial fibres were stained red. The NM and DM myocardial fibres in the I/R group displayed a disorder arrangement and large amounts of collagen deposition compared with sham group. Silencing of AK139328 alleviated the effects of I/R on myocardial fibres (Figure 3B). Evans Blue/TTC staining reflected that I/R led to myocardial infarction in NM and DM, and the infarct size in the latter group was significantly larger. Low expression of lncRNA AK139328 remarkably reduced the myocardial infarct size of DM, which relieved the injury of I/R as well (Figure 3C). Results of Western blot presented that the level of α‐SMA in NM and DM myocardial tissues was reduced after I/R, but knockdown of AK139328 relieved this effect and recovered the level of α‐SMA (Figure 3D). Thus, silencing of lncRNA AK139328 conduces to better recovery after I/R in diabetic models.

### 3.4 Knockdown of lncRNA AK139328 inhibited cardiomyocyte autophagy and apoptosis of DM

**Figure 4:**
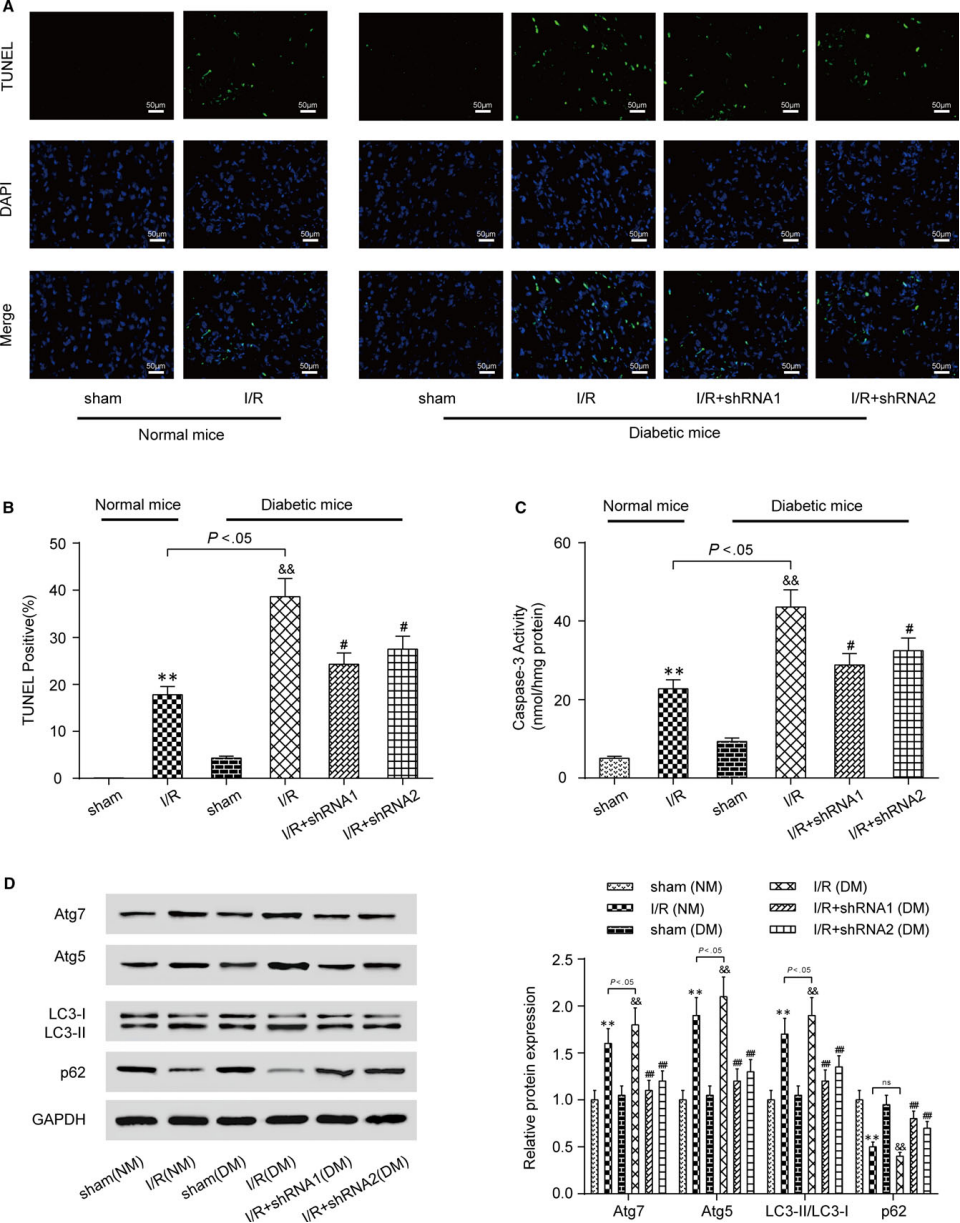
Knockdown of lncRNA AK139328 inhibited cardiomyocyte apoptosis and autophagy of DM. A and B, Knockdown of lncRNA AK139328 inhibited cardiomyocyte apoptosis in DM. C, Cardiomyocyte apoptosis in MI/R (DM) group was significantly higher than that in MI/R (NM) group. D, MIRI promoted the expression of Atg7, Atg5 and LC3‐II/LC3‐I and inhibited the expression of p62, whereas knockdown of lncRNA AK139328 recovered protein expression level. **P < .01, compared with sham (NM) group, ^&&^ *P* < .01, compared with sham (DM) group, and ^#^ *P* < .05, ^##^ *P* < .01, compared with I/R (DM) group

The result of TUNEL staining indicated that I/R caused cardiomyocyte apoptosis in both NM and DM, and the apoptosis rate of DM cells significantly exceeded that of NM cells. Knockdown of lncRNA AK139328 significantly inhibited cardiomyocyte apoptosis in DM (Figure 4A,B). Caspase‐3 activity assay exhibited the same outcome (Figure 4C). MIRI promoted the expression of Atg7, Atg5 and LC3‐II/LC3‐I and inhibited the expression of p62, whereas knockdown of lncRNA AK139328 reversed the above variation in protein expression levels marking cell autophagy (Figure 4D). To sum up, knockdown of lncRNA AK139328 protects myocardial cells from apoptosis and autophagy.

### 3.5 LncRNA AK139328 modulated miR‐204‐3p directly

**Figure 5:**
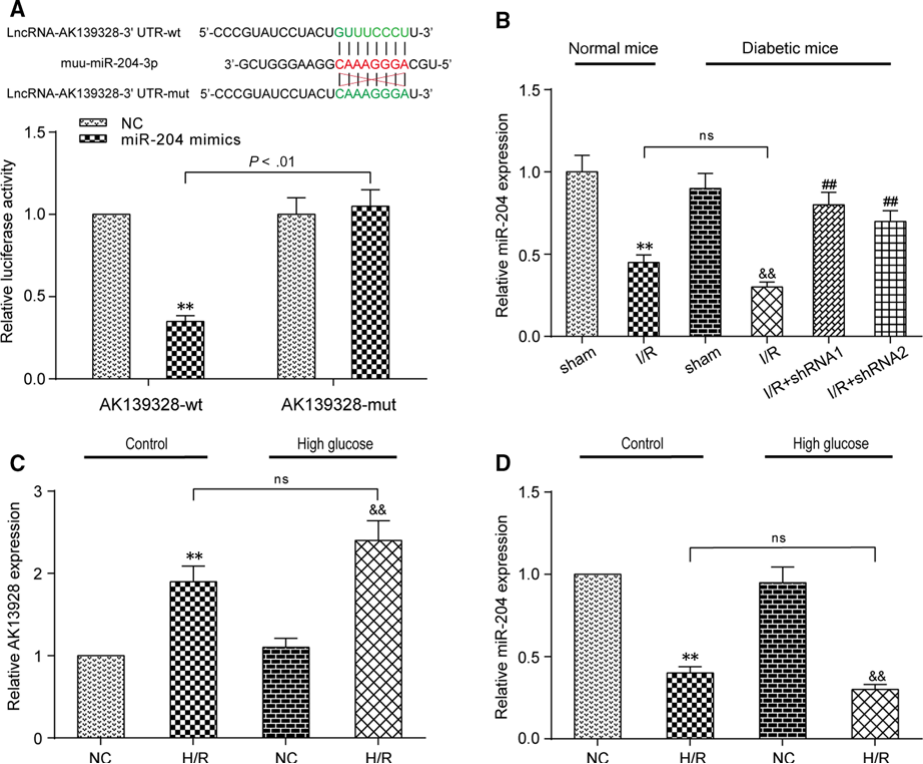
LncRNA AK139328 targeted and modulated miR‐204‐3p. A, Bioinformatics prediction and dual‐luciferase reporter assay. MiR‐204‐3p‐binding site in lncRNA AK139328 wild‐type form (AK139328‐wt) and the mutated form (AK139328‐mut) were shown in the upper panel. HEK293T cells were transfected with miR‐204‐3p mimics or mimic‐NC and then transfected with the luciferase constructs of AK139328‐wt or AK139328‐mut. The luciferase activity was analysed. B, The expression of miR‐204‐3p was inhibited in I/R (NM) group and I/R (DM) group and the expression of miR‐204‐3p rose in I/R + shRNA groups. **P < .01, compared with sham (NM) group, ^&&^ *P* < .01, compared with sham (DM) group, and ^##^ *P* < 0.01, compared with I/R (DM) group. C, Relative AK139328 expression was promoted in hypoxia/reoxygenation (H/R) (control) group and H/R (high‐glucose) group, compared with NC groups. **P < .01, compared with NC (control) group, and ^&&^ *P* < .01, compared with NC (high‐glucose) group. D, Relative miR‐204‐3p expression was inhibited in H/R (control) group and H/R (high‐glucose) group, compared with NC groups. **P < .01, compared with NC (control) group, and ^&&^ *P* < .01, compared with NC (high‐glucose) group

First, we produced a luciferase construct of AK139328 (AK139328‐wt) and a mutated form (AK139328‐mut) (Figure 5A). Luciferase assay revealed that miR‐204‐3p mimic could suppress the luciferase activity of AK139328‐wt, but it had less effect on the mutated form of AK139328 (Figure 5A). These results revealed that AK139328 may interact with miR‐204‐3p by this putative binding site. RT‐qPCR results indicated that the expression of miR‐204‐3p was remarkably inhibited in I/R (NM) group and I/R (DM) group, compared with sham groups. The expression of miR‐204‐3p was up‐regulated in I/R + shRNA groups (Figure 5B). In H/R injury model construction, transfection efficiency of shRNA1‐AK139328 group, miR‐204‐3p mimic group and miR‐204‐3p inhibitor group was tested with RT‐qPCR assay. Relative AK139328 expression was promoted in H/R (control) group and H/R (high‐glucose) group, compared with NC groups (Figure 5C). On the contrary, relative miR‐204‐3p expression was inhibited in H/R (control) group and H/R (high‐glucose) group, compared with NC groups (Figure 5D). Therefore, miR‐204‐3p was the direct target of lncRNA AK139328.

### 3.6 AK139328 knockdown and miR‐204‐3p overexpression relieved H/R injury via inhibiting autophagy

**Figure 6:**
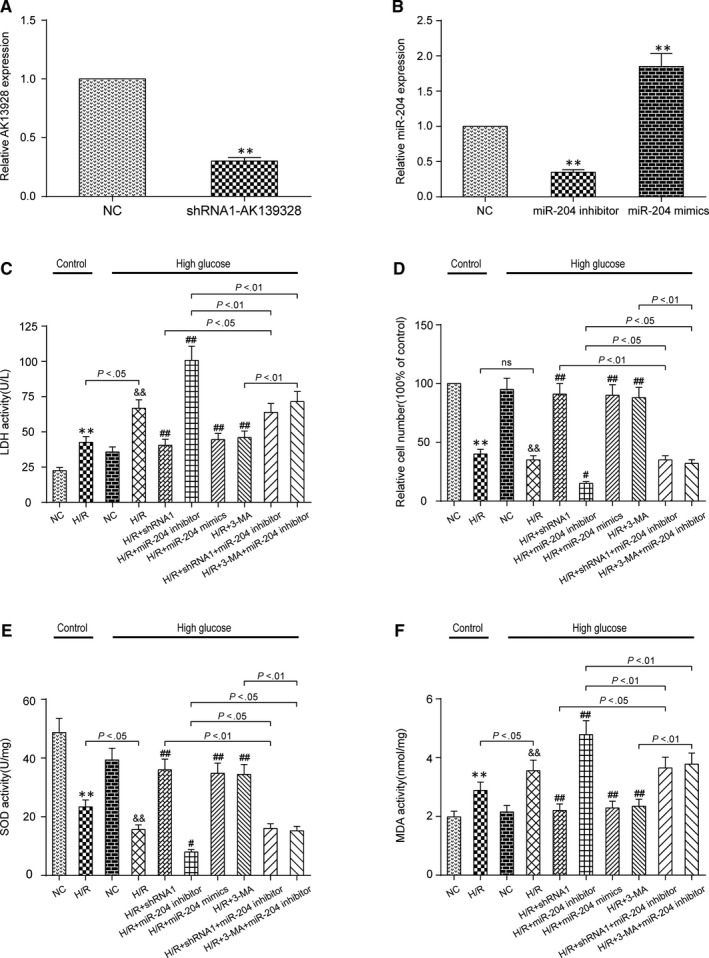
LncRNA AK139328 knockdown and miR‐204‐3p overexpression relieved hypoxia/reoxygenation (H/R) injury in high‐glucose environment. A, Relative AK139328 expression was reduced in shRNA1‐AK139328 group. **P < .01, compared with NC group. B, Relative miR‐204‐3p expression was promoted in miR‐204‐3p mimic group and reduced in miR‐204‐3p inhibitor group. **P < .01, compared with NC group. C, The expression of LDH increased after H/R injury. shRNA1, miR‐204 mimics and 3‐MA reduced the expression of LDH, which relieved H/R injury, while miR‐204 inhibitor promoted it. D, H/R injury significantly reduced the number of viable cells, which was lower in H/R + miR‐204 inhibitor group and higher in H/R + shRNA1 group, H/R + miR‐204 mimic group and H/R + 3‐MA group. E, SOD concentration was inhibited after H/R injury both in control group and in high‐glucose group. SOD concentration was recovered in H/R + shRNA1 group, H/R + miR‐204 mimic group and H/R + 3‐MA group but inhibited in H/R + miR‐204 inhibitor group. F, MDA concentration was promoted after H/R injury both in control group and in high‐glucose group. MDA concentration was promoted in H/R + miR‐204 inhibitor group and recovered in H/R + shRNA1 group, H/R + miR‐204 mimic group and H/R + 3‐MA group. **P < .01, compared with NC (control) group, ^&&^ *P* < .01, compared with NC (high‐glucose) group, and ^#^ *P* < .05, ^##^ *P* < .01, compared with H/R (high‐glucose) group

**Figure 7:**
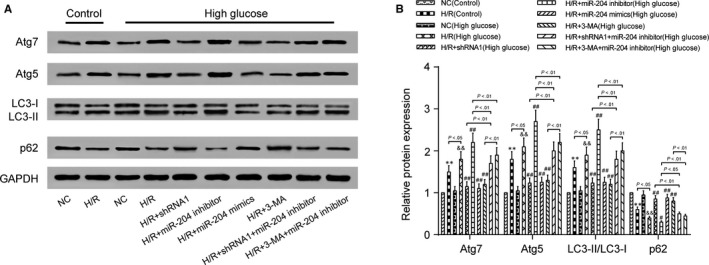
AK139328 knockdown and miR‐204‐3p overexpression relieved H/R injury via inhibiting cardiomyocyte autophagy. A and B, The expression of Atg7, Atg5 and LC3‐II/LC3‐I was reduced in H/R + shRNA1 group, H/R + miR‐204 mimic group and H/R + 3‐MA group and promoted in H/R + miR‐204 inhibitor group. **P < .01, compared with NC (control) group, ^&&^ *P* < .01, compared with NC (high‐glucose) group, and ^#^ *P* < .05, ^##^ *P* < .01, compared with H/R (high‐glucose) group

Relative AK139328 expression was reduced in shRNA1‐AK139328 group (Figure 6A). Relative miR‐204‐3p expression was increased in miR‐204‐3p mimic group, while reduced in miR‐204‐3p inhibitor group (Figure 6B). The above results all indicated successful transfection during secondary grouping. Autophagy inhibitor 3‐MA was utilized to investigate the role of autophagy in H/R injury. The concentration of LDH increased after H/R injury, and in high‐glucose group, the increase was significantly larger than that in control group. Considering high‐glucose environment, shRNA1, miR‐204‐3p mimics and 3‐MA reduced the concentration of LDH, which relieved H/R injury, while miR‐204‐3p inhibitor promoted it. shRNA1 and 3‐MA attenuated the effect of miR‐204‐3p inhibitor on LDH concentration (Figure 6C). The results of MTT assay showed that H/R injury significantly reduced the number of viable cells, and the reduction was more significant in H/R + miR‐204‐3p inhibitor group, but was impaired in H/R + shRNA1 group, H/R + miR‐204‐3p mimic group and H/R + 3‐MA group. Cotransfection with shRNA1 or 3‐MA reversed the effect of miR‐204‐3p inhibitor (Figure 6D). SOD concentration was inhibited after H/R injury both in control group and in high‐glucose group, and this repression was stronger in high‐glucose group. It was recovered in H/R + shRNA1 group, H/R + miR‐204‐3p mimic group and H/R + 3‐MA group. The inhibition was aggravated in H/R + miR‐204‐3p inhibitor group, which was moderated on transfection of shRNA1 or 3‐MA (Figure 6E). High‐glucose environment enhanced MDA concentration, under which miR‐204‐3p mimics, shRNA1 and 3‐MA controlled this increase, but miR‐204‐3p inhibitor acted the reverse way and tended to be negatively influenced by the latter two (Figure 6F). Western blot assay indicated that the expression of Atg7, Atg5 and LC3‐II/LC3‐I was promoted in H/R groups compared with NC groups in both control and high‐glucose groups, while p62 expression was inhibited. The expression of Atg7, Atg5 and LC3‐II/LC3‐I was reduced in H/R + shRNA1 group, H/R + miR‐204‐3p mimic group and H/R + 3‐MA group and promoted in H/R + miR‐204‐3p inhibitor group. shRNA1 and 3‐MA repressed miR‐204‐3p inhibitor‐induced increase. Contrary to Atg7, Atg5 and LC3‐II/LC3‐I, the level of p62, which was negatively associated with autophagy, acted likewise but towards an opposite direction (Figure 7A,B). Therefore, lncRNA AK139328 relieved H/R injury through inhibiting cardiomyocyte autophagy via regulating miR‐204‐3p.

## 4 DISCUSSION

Autophagy has been reported by the recent literature to be protective for myocardial I/R, and there are increasing studies on autophagy in interaction with apoptosis in ischaemic myocardium.^18^ For instance, Zheng et al investigated berbamine pre‐treatment on protecting I/R injury in hearts on the basis of autophagy mechanism.^19^ Ma et al emphasized ALDH2 as therapeutic target for MIRI and it prompting autophagy during ischaemia and remained elevated autophagy at reperfusion.^20^ Yao et al argued the MIRI preventive role of vitamin D receptor in that its overexpression repressed abnormal apoptosis and autophagy.^21^ Our work figured out that knockdown of lncRNA AK139328 relieved the damage of I/R to myocardium tissue and reduced infarction size. We identified miR‐204‐3p as the direct target of lncRNA AK139328 and negatively regulated autophagy and apoptosis both in animal normal or in diabetic models and *in vitro* experiments of normal or high‐glucose condition. We concluded that by targeting miR‐204‐3p, silencing AK139328 mitigated MIRI injury via regulation of autophagy.

Diabetes of either type increased the danger of MIRI. As was argued by Volz et al, HMGB1 up‐regulation leads the underlying mechanism of type 1 diabetes mellitus being in positive correlation with ischaemic cardiomyopathy.^22^ Our *in vivo* experiments revealed the same relationship as demonstrated by larger infarction size and damaged areas in type 2 diabetic mice. On the other hand, Przyklenk et al found that the relief of after conditioning on myocardial ischaemia was blocked in both type 1 and type 2 mice hearts.^23^ For further investigation of MIRI‐relating diabetes, our future experiments could be designed for lncRNA AK139328 treatment on diabetic (type 1/2) or normal models to figure out its regulation on autophagy.

lncRNA AK139328 was recently highlighted for its involvement in mice I/R injury and cell apoptosis of livers, and we made the first attempt in speculating its function in myocardial I/R.^9^ Similar engagement of other lncRNAs has already been probed for. Vausort et al measured different expression levels of 5 lncRNAs in patients of acute myocardial ischaemia and hypothesized their being therapeutic targets.^24^ In our results, lncRNA AK139328 was selected for its highest up‐regulation by microarray screening as well as RT‐qPCR result corroboration. We also affirmed AK139328's correlation with myocardial cell autophagy. For previous documentation regarding lncRNAs regulating autophagy in myocardial infarction, Wang et al demonstrated that lncRNA APF manipulated myocardial infarction and autophagy.^25^ Yet whether and how lncRNAs are engaged in autophagy in other diseases and tissues remained prospective for further discussion.

MiR‐204‐3p has been widely accepted as a tumour suppressor. Chen et al stated that miR‐204‐3p demoted glioma cell proliferation by enhancing glioma cell apoptosis.^26^ Cui et al found overexpression of miR‐204‐3p in hepatocellular carcinoma (HCC) tumour endothelial cells (TECs) significantly inhibited the proliferation of HCC TECs and promoted apoptosis.^27^ In our present results, miR‐204‐3p acted as the direct target of lncRNA AK139328 and modified myocardial cell autophagy during MIRI recovery. In spite of being regulated by distinct lncRNAs, chances are that miR‐204‐3p served as autophagy regulator to be the therapeutic target of various kinds.

Limitations still exist in this study. Firstly, as all our assays were performed in mice, the experimental results might not be directly extrapolated to humans. For future experiments, we may introduce tests on other animal models such as rates. We also required further identification and confirmation of the precise mechanisms underlying AK139328 and miR‐204‐3p. Above all, we made a successful attempt in identifying lncRNA AK139328 as MIRI‐influencing factor.

Ongoing researches showed that lncRNAs and miRNAs played a powerful role in almost all cellular events including myocardial I/R injury. Higher levels of apoptosis and autophagy have been detected after MIRI. AK139328 and miR‐204‐3p have been indicated to modulate such cellular processes. Knockdown of AK139328 attenuated myocardial ischaemia/reperfusion injury and inhibited cardiomyocyte autophagy and apoptosis in DM. It was confirmed that AK139328 directly modulated miR‐204‐3p, thereby modulating apoptosis and autophagy.

## CONFLICT OF INTEREST STATEMENT

The authors confirm that there are no conflict of interests.
