# Corrigendum

**DOI:** 10.1111/jcmm.16258

**Published:** 2021-02-10

In the published article by Yu et al., an error was found in Figure 4A‐B. Upon clarification from the authors and investigation of the images in the paper, it has been decided to proceed with a corrigendum. The authors confirm all results and conclusions of this article remain unchanged. The corrected figure is shown below:

**Figure:**
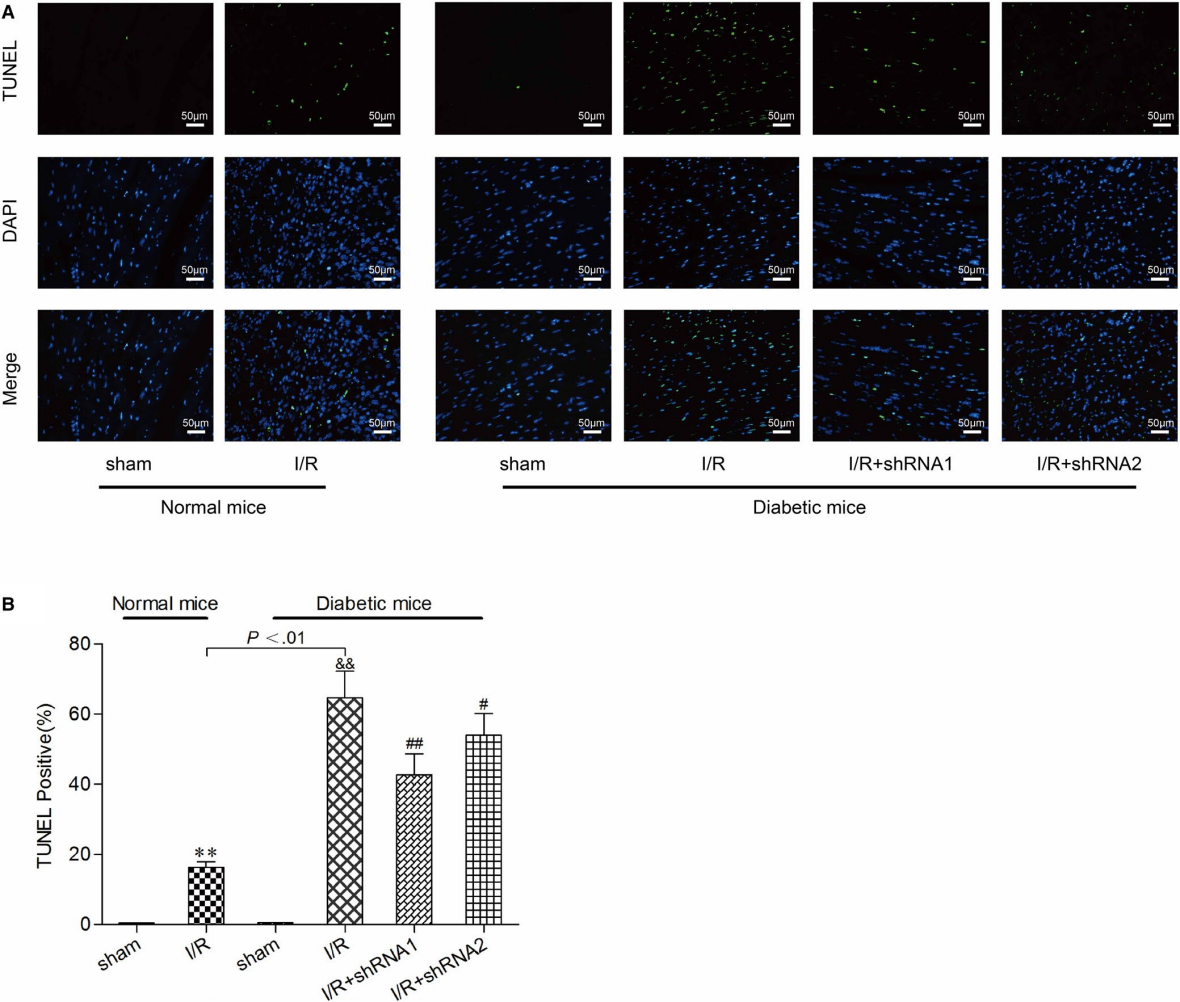
Knockdown of lncRNA AK139328 inhibited cardiomyocyte apoptosis and autophagy of DM. A and B, Knockdown of lncRNA AK139328 inhibited cardiomyocyte apoptosis in DM. Cardiomyocyte apoptosis in MI/R (DM) group was significantly higher than that in MI/R (NM) group. D, MIRI promoted the expression of Atg7, Atg5 and LC3‐II/LC3‐I and inhibited the expression of p62, whereas knockdown of lncRNA AK139328 recovered protein expression level. **P < .01, compared with sham (NM) group, &&*P* < .01, compared with sham (DM) group, and #*P* < .05, ##*P* < .01, compared with I/R (DM) group
