# Supplementary figures and images for: Knockdown of lncRNA AK139328 alleviates myocardial ischaemia/reperfusion injury in diabetic mice via modulating miR‐204‐3p and inhibiting autophagy
Source: J Cell Mol Med. 2018 Jul 25;22(10):4886–98. doi: 10.1111/jcmm.13754 (PMC6156366; doi:10.1111/jcmm.13754)

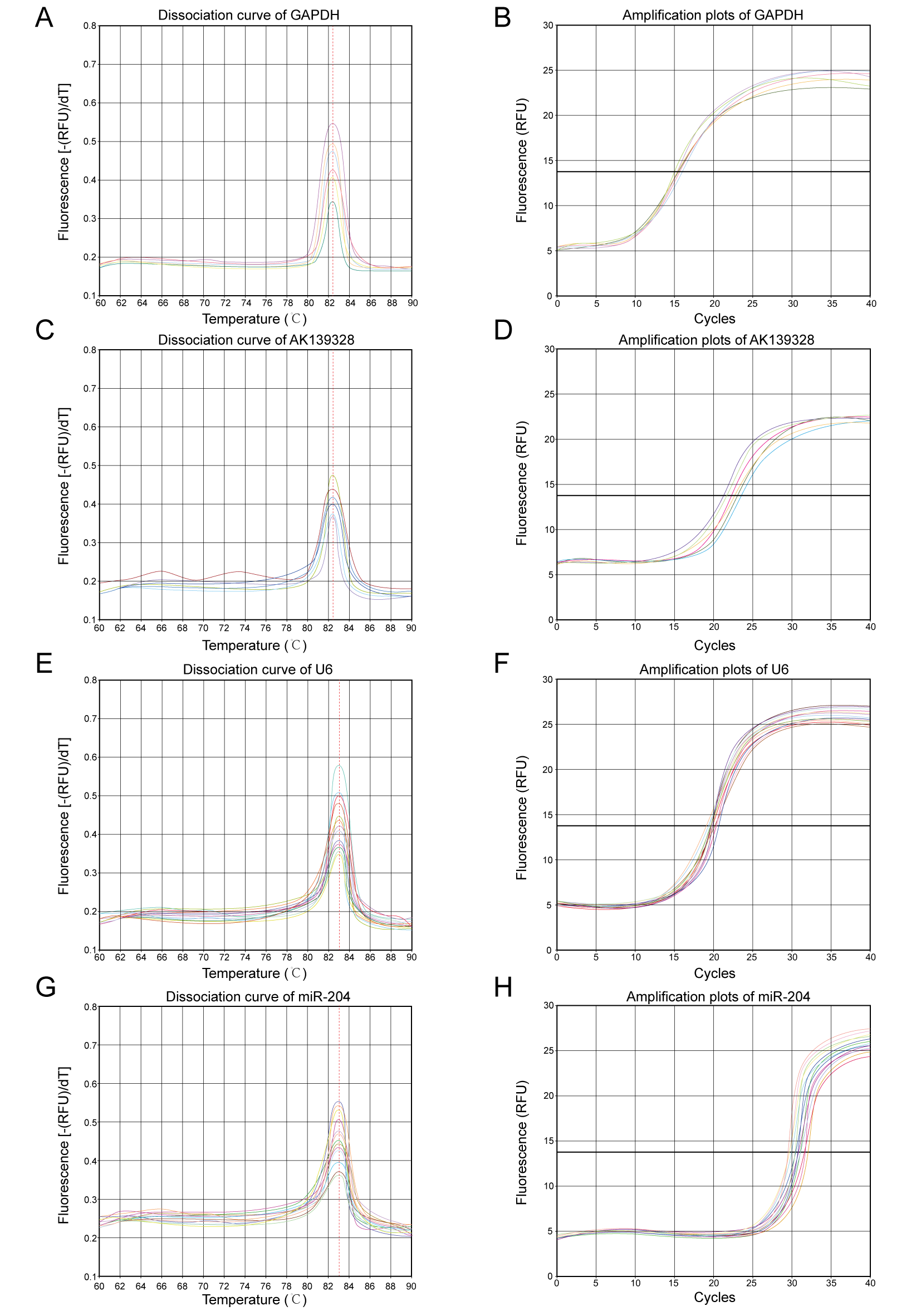

Supplement: Supplementary file 1 [file JCMM-22-4886-s001.tif]
