# Supplementary material for: Knockdown of lncRNA AK139328 alleviates myocardial ischaemia/reperfusion injury in diabetic mice via modulating miR‐204‐3p and inhibiting autophagy
Source: J Cell Mol Med. 2018 Jul 25;22(10):4886–98. doi: 10.1111/jcmm.13754 (PMC6156366; doi:10.1111/jcmm.13754)
Supplement: Supplementary file 2 [file JCMM-22-4886-s002.docx]

**Table 1. The biological characteristics of C57BL/KsJ db/+ and C57BL/KsJ db/db mice**

| **Items** | **C57BL/KsJ db/+** | **C57BL/KsJ db/db** |
| --- | --- | --- |
| Weight (g) | 23.4 ± 2.67 | 25.8 ± 1.95 |
| Blood glucose (mmol/L) | 8.12 ± 2.34 | 22.38 ± 3.46^**^ |
| Serum insulin (ng/mL) | 1.21 ± 0.56 | 15.37 ± 2.28^**^ |

^**^ *P* < 0.05 compared with C57BL/KsJ db/+ mice
