# Supplementary material for: Knockdown of lncRNA AK139328 alleviates myocardial ischaemia/reperfusion injury in diabetic mice via modulating miR‐204‐3p and inhibiting autophagy
Source: J Cell Mol Med. 2018 Jul 25;22(10):4886–98. doi: 10.1111/jcmm.13754 (PMC6156366; doi:10.1111/jcmm.13754)
Supplement: Supplementary file 3 [file JCMM-22-4886-s003.docx]

**Table 2. Primers for qRT-PCR**

| **Gene** | **Sequence（5’-3’）** |
| --- | --- |
| LncRNA-AK139328 |  |
| Forward primer | CCAGTTCTTGGTCCTGGTGT |
| Reverse primer | GTGTCTGCAACCCGATAGGT |
| LncRNA-AK028326 |  |
| Forward primer | GGCTTATGGGAGGGGCAAAT |
| Reverse primer | GAGGCATGAAATCACCCCCA |
| LncRNA-Malat1 |  |
| Forward primer | GGACTTGAGCTGAGGTGCTT |
| Reverse primer | GCTTCACCAAACTGGCTTCG |
| LncRNA-Eif4a2 |  |
| Forward primer | GCCTCAGCAAGCAATCATCC |
| Reverse primer | TGAGCCTTTGTGCAACTGTG |
| LncRNA-Gomafu |  |
| Forward primer | GCCCATTCTCTGATCTCGGG |
| Reverse primer | CCGTTAGTTAAGGGGCAGCA |
| LncRNA-Vps13d |  |
| Forward primer | CCAGACTCGGCTCCTGTTAG |
| Reverse primer | ACTCCCTGCCCCACAAACTA |
| LncRNA-Slco6d1 |  |
| Forward primer | GCTGGCTGCTTTCTAACAGG |
| Reverse primer | ATTCCATGGAGACACTGCCC |
| LncRNA-Aasdh |  |
| Forward primer | GCAAAGGCAACAGAACCCAG |
| Reverse primer | TCACCGTTCCAAGTAGTGGC |
| miR-204-3p |  |
| Forward primer | TGTTGCAGTGAGGGCAAGAA |
| Reverse primer | GACCCTGGTTGCTTCAAGGA |
| GAPDH |  |
| Forward primer | AGCCACATCGCTCAGACAC |
| Reverse primer | GCCCAATACGACCAAATCC |
| U6 |  |
| Forward primer | ATTGGAACGATACAGAGAAGATT |
| Reverse primer | GGAACGCTTCACGAATTTG |
