# Supplementary material for: Knockdown of lncRNA AK139328 alleviates myocardial ischaemia/reperfusion injury in diabetic mice via modulating miR‐204‐3p and inhibiting autophagy
Source: J Cell Mol Med. 2018 Jul 25;22(10):4886–98. doi: 10.1111/jcmm.13754 (PMC6156366; doi:10.1111/jcmm.13754)
Supplement: Supplementary file 4 [file JCMM-22-4886-s004.docx]

**Table 3.** **ShRNA sequence of lncRNA-AK139328**

| **Gene** | **Sequence（5’-3’）** |
| --- | --- |
| **shRNA1** |  |
| Top Strand | CACCGGAAACTCAGCTATCACATGCCGAAGCATGTGATAGCTGAGTTTCC |
| Bottom Strand | AAAAGGAAACTCAGCTATCACATGCTTCGGCATGTGATAGCTGAGTTTCC |
| **shRNA2** |  |
| Top Strand | CACCGCAGCAGAAAGACATGTTTGGCGAACCAAACATGTCTTTCTGCTGC |
| Bottom Strand | AAAAGCAGCAGAAAGACATGTTTGGTTCGCCAAACATGTCTTTCTGCTGC |
| **sh-NC** |  |
| Top Strand | CACCTTCTCCGAACGTGTCACGTTTCAAGAGAACGTGACACGTTCGGAGAATTTTTTG |
| Bottom Strand | GATCCAAAAAATTCTCCGAACGTGTCACGTTCTCTTGAAACGTGACACGTTCGGAGAA |
